# Antipsychotics and Sleep Architecture: Where Does Lurasidone Fit?

**DOI:** 10.7759/cureus.113943

**Published:** 2026-08-04

**Authors:** Binh Pham, Sakeena Siddiq

**Affiliations:** 1 Sleep Medicine, Southwest Healthcare Medical Education Consortium, Temecula, USA; 2 Psychiatry, Western University of Health Sciences, Pomona, USA

**Keywords:** circadian rhythm, clinical pharmacology, insomnia disorder, lurasidone, psychiatric pharmacology, rem and nrem sleep, second-generation antipsychotics, sleep pharmacology, sleep-wake cycle, sleep-wake patterns

## Abstract

Sleep disturbances are highly prevalent in schizophrenia, bipolar disorder, and other psychiatric disorders and are associated with increased symptom burden, impaired functioning, and reduced quality of life. Although second-generation antipsychotics frequently affect sleep, their effects vary according to receptor-binding profile and should not be viewed solely as a consequence of sedation. Lurasidone is distinguished by antagonism at D2, 5-HT2A, and 5-HT7 receptors with minimal affinity for histamine H1 and muscarinic M1 receptors, a pharmacologic profile associated with relatively low intrinsic sedation. This narrative review summarizes the neurobiology of sleep, examines receptor-based hypotheses through which lurasidone may influence sleep, and reviews the available preclinical and clinical evidence regarding its effects on sleep continuity and sleep architecture. Limited human evidence suggests that lurasidone may improve objective measures of sleep continuity, whereas preclinical findings suggest possible effects on rapid eye movement and non-rapid eye movement sleep. However, direct human evidence supporting modification of sleep architecture remains limited, and existing phase-advance data should not be interpreted as evidence of altered circadian physiology. Dedicated sleep studies remain few, and long-term data using polysomnography, actigraphy, circadian biomarkers, and validated measures of daytime functioning are lacking. Further comparative studies are needed to determine whether lurasidone provides clinically meaningful sleep-related benefits beyond improvements attributable to treatment of the underlying psychiatric disorder.

## Introduction and background

Sleep disturbances are among the most prevalent and clinically significant symptoms encountered in psychiatry and sleep medicine. Numerous psychotropic medications influence sleep, including hypnotics, anxiolytics, sedating antidepressants, and antipsychotics. Although antipsychotics are prescribed primarily for schizophrenia, bipolar disorder, and other psychiatric illnesses, their effects on sleep frequently influence medication selection in clinical practice. Clinicians must therefore consider not only psychiatric symptom control but also treatment-related effects on sleep quality, daytime alertness, cognition, and overall functioning [1,2].

Sleep abnormalities are highly prevalent among individuals with schizophrenia and bipolar disorder and commonly include insomnia, fragmented sleep, circadian rhythm disruption, and alterations in sleep architecture. These disturbances are associated with greater symptom burden, impaired cognition, poorer functional outcomes, reduced quality of life, and an increased risk of relapse. Accordingly, the assessment and treatment of sleep disturbance represent important components of comprehensive psychiatric care rather than secondary considerations limited to the management of psychosis or mood symptoms [2,3].

Normal sleep is organized into recurring cycles of non-rapid eye movement (NREM) and rapid eye movement (REM) sleep that repeat approximately every 90 to 120 minutes throughout the night. NREM sleep consists of stages N1, N2, and N3, representing progressively deeper levels of sleep. N1 is the transitional stage between wakefulness and sleep; N2 accounts for the greatest proportion of total sleep time and is characterized by sleep spindles and K-complexes; whereas N3, or slow-wave sleep, is associated with restorative physiologic processes, immune regulation, and memory consolidation. REM sleep is characterized by cortical activation, skeletal muscle atonia, rapid eye movements, and vivid dreaming and plays an important role in emotional regulation, procedural learning, and memory processing. Preservation of normal sleep architecture, in addition to adequate sleep duration and continuity, is therefore essential for healthy cognitive and physiologic functioning [4,5].

Psychiatric disorders may disrupt multiple components of sleep architecture, including sleep continuity, slow-wave sleep, REM sleep, and circadian organization. Likewise, antipsychotic medications exert heterogeneous effects on sleep that extend beyond sedation alone. Some agents facilitate sleep primarily through histamine H1 receptor antagonism, whereas others influence sleep continuity and sleep architecture through serotonergic, dopaminergic, muscarinic, and adrenergic mechanisms. Consequently, improvements in subjective sleep quality or total sleep time should not be assumed to represent normalization of sleep architecture or restoration of physiologic sleep. Recent systematic reviews further demonstrate considerable variability in the effects of second-generation antipsychotics on objective sleep measures, underscoring the importance of distinguishing sedation from meaningful improvements in sleep physiology [6-8].

Among second-generation antipsychotics, lurasidone has generated interest because of its relatively low intrinsic sedative burden compared with more strongly antihistaminergic agents [1]. This characteristic raises an important clinical question: where does lurasidone fit among antipsychotics with respect to sleep continuity, sleep architecture, circadian regulation, and overall sleep quality? This narrative review examines the neurobiology of sleep, the mechanisms through which antipsychotics influence sleep, the available preclinical and clinical evidence regarding lurasidone, and its potential position relative to other second-generation antipsychotics. Although previous reviews have broadly examined the effects of second-generation antipsychotics on sleep, few have specifically synthesized the mechanistic and emerging clinical evidence regarding lurasidone, particularly its unique receptor pharmacology and potential implications for sleep continuity and sleep architecture.

The classification, principal physiologic characteristics, and neurobiology of normal sleep stages, together with the potential effects of second-generation antipsychotics on these stages, are summarized in Table 1.

**Table 1:** Normal sleep architecture: classification, neurobiology, and potential effects of second-generation antipsychotics The effects of second-generation antipsychotics summarized in this table are qualitative and based on the cited literature. Individual effects may vary according to medication, receptor-binding profile, dosage, treatment duration, psychiatric diagnosis, concomitant therapies, and study methodology. 5-HT, 5-hydroxytryptamine; GABA, gamma-aminobutyric acid; H1, histamine H1 receptor; NREM, non-rapid eye movement; REM, rapid eye movement.

Sleep stage	Principal physiologic characteristics	Relevant neurobiology	Potential effects of second-generation antipsychotics
Wakefulness	Sustained environmental awareness, behavioral responsiveness, and relatively high cortical activity	Wakefulness is maintained by the ascending arousal system, including histaminergic, noradrenergic, dopaminergic, serotonergic, cholinergic, and orexinergic pathways [1,4].	Histamine H1 receptor antagonism may reduce arousal and produce somnolence, whereas dopaminergic and serotonergic effects may influence activation, vigilance, and daytime functioning [1,7,8].
N1 sleep	Lightest stage of NREM sleep; transition between wakefulness and sleep; individuals remain readily arousable	Progressive reduction in ascending arousal activity with increasing influence of GABAergic sleep-promoting neurons [4]	More sedating agents may shorten sleep latency and facilitate sleep initiation, although this may primarily reflect antihistaminergic sedation rather than normalization of sleep architecture [7,8].
N2 sleep	Characterized by sleep spindles and K-complexes; constitutes the largest proportion of total sleep time	Thalamocortical networks generate sleep spindles and K-complexes while reduced arousal system activity promotes stable NREM sleep [4].	Some second-generation antipsychotics may improve sleep continuity or increase stage N2 sleep; however, effects on objective sleep measures remain variable among agents and study populations [7,8].
N3 sleep (slow-wave sleep)	Deepest stage of NREM sleep; characterized by high-amplitude, low-frequency delta activity; associated with physical restoration and memory consolidation	Cortical synchronization and GABAergic sleep-promoting activity contribute to the generation and maintenance of slow-wave sleep [4].	Selected second-generation antipsychotics have been associated with increased slow-wave sleep, although this effect is not consistent across medications [7,8].
REM sleep	Characterized by rapid eye movements, cortical activation, vivid dreaming, and skeletal muscle atonia	REM sleep is associated with increased cholinergic activity and reduced serotonergic and noradrenergic neuronal activity [4].	Effects on REM sleep duration and latency are heterogeneous and vary according to receptor-binding profile, medication, dose, study population, and study design [7,8].

## Review

Literature search strategy

A narrative literature review was conducted to synthesize the available evidence regarding the effects of lurasidone on sleep architecture and its potential role in sleep regulation. Electronic searches were performed using PubMed and Google Scholar to identify relevant English-language publications. Search terms included combinations of "lurasidone", "sleep", "sleep architecture", "polysomnography", "rapid eye movement (REM) sleep", "non-rapid eye movement (NREM) sleep", "slow-wave sleep", "circadian rhythm", "second-generation antipsychotics", "atypical antipsychotics", "sleep physiology", and "5-HT7 receptor."

Studies were selected based on their relevance to one or more of the following domains: (1) the neurobiology and physiology of sleep; (2) the pharmacologic mechanisms through which antipsychotic medications influence sleep architecture; (3) the receptor pharmacology of lurasidone; (4) clinical investigations evaluating sleep outcomes associated with lurasidone treatment; and (5) comparative studies examining the effects of second-generation antipsychotics on sleep. Relevance was assessed by the authors according to scientific quality, direct applicability to the objectives of this review, and the contribution of each study to the understanding of lurasidone, sleep physiology, or antipsychotic pharmacology. Studies were selected by the authors based on these relevance criteria. Human and preclinical studies were included when they provided meaningful mechanistic or clinical insights, whereas publications lacking direct relevance to the review objectives or containing insufficient methodological or clinical information were excluded. Additional articles were identified through manual review of the reference lists of relevant publications to improve the comprehensiveness of the literature search.

Given the limited number of studies directly evaluating lurasidone using objective sleep measures such as polysomnography, evidence from mechanistic investigations, receptor pharmacology, animal models, post hoc analyses, and the broader literature on second-generation antipsychotics was incorporated to provide context and better characterize the potential effects of lurasidone on sleep architecture. This review is intended as a narrative synthesis of the current literature rather than a systematic review or meta-analysis. Accordingly, no formal risk-of-bias assessment was performed, and study selection was not based on predefined systematic review procedures. The findings should therefore be interpreted as a qualitative synthesis of the available evidence rather than a quantitative or comprehensive systematic evaluation. Because this article was designed as a narrative review rather than a systematic review, the literature search and study selection procedures were not designed to support full reproducibility and may be subject to selection bias.

Neurobiology of sleep and mechanisms of antipsychotic action

Sleep is an actively regulated neurophysiologic process governed by the interaction of homeostatic sleep pressure (Process S) and the circadian timing system (Process C), which together coordinate transitions between wakefulness, NREM sleep, and REM sleep [1-4]. Rather than representing passive states, sleep and wakefulness are maintained by dynamic interactions among hypothalamic, brainstem, thalamic, and cortical networks that continuously balance arousal and sleep-promoting influences. Disruption of these regulatory mechanisms contributes to many psychiatric disorders and frequently manifests as insomnia, hypersomnolence, circadian rhythm disturbances, and alterations in sleep architecture [5,6].

The transition between sleep and wakefulness is mediated by reciprocal interactions between sleep-promoting neurons within the ventrolateral preoptic nucleus (VLPO) and wake-promoting nuclei of the ascending arousal system. GABAergic neurons of the VLPO inhibit multiple arousal centers, including histaminergic neurons of the tuberomammillary nucleus, noradrenergic neurons of the locus coeruleus, serotonergic neurons of the dorsal raphe nucleus, cholinergic neurons of the basal forebrain, and dopaminergic pathways originating from the ventral tegmental area. Conversely, these arousal systems suppress VLPO activity during wakefulness, forming a bistable "flip-flop" switch that promotes stable transitions between sleep and wake. Orexin-producing neurons within the lateral hypothalamus further stabilize this circuitry by reinforcing wakefulness and preventing inappropriate transitions between behavioral states [3,9].

Many of the neurotransmitter systems responsible for regulating sleep are also the primary pharmacologic targets of second-generation antipsychotics. Histamine and norepinephrine promote wakefulness, acetylcholine plays a central role in cortical activation and REM sleep, serotonin (5-hydroxytryptamine (5-HT)) contributes to circadian regulation and REM modulation, and dopamine participates in arousal and behavioral activation [1,3,4,10]. Consequently, modulation of these neurotransmitter systems may influence not only sleep initiation and maintenance but also sleep architecture, REM expression, slow-wave sleep, and next-day alertness.

The effects of second-generation antipsychotics on sleep therefore vary according to their individual receptor-binding profiles rather than class effects alone. Histamine H1 receptor antagonism is strongly associated with sedation and reduced sleep latency, whereas muscarinic and α1-adrenergic receptor antagonism further contribute to sedation and other adverse effects. In contrast, serotonergic mechanisms appear to exert more direct effects on sleep architecture. Antagonism of the 5-HT2A receptor has been associated with improved sleep continuity and increased slow-wave sleep, whereas the 5-HT7 receptor has emerged as an important regulator of circadian rhythmicity, REM sleep, and sleep-wake cycling [1,10-12]. Together, these observations suggest that differences in receptor-binding affinity are likely responsible for the heterogeneous effects of second-generation antipsychotics on subjective sleep quality and objective polysomnographic measures.

Lurasidone and sleep

Lurasidone is distinguished from many second-generation antipsychotics by its receptor-binding profile. It exhibits high affinity for dopamine D2, 5-HT2A, and 5-HT7 receptors while demonstrating minimal affinity for histamine H1 and muscarinic M1 receptors [1,13]. Unlike antipsychotics such as clozapine, olanzapine, and quetiapine, whose sedative properties are largely mediated through potent histamine H1 receptor antagonism, lurasidone produces relatively little intrinsic sedation. This pharmacologic distinction raises the possibility that improvements in sleep may occur through modulation of sleep-regulatory pathways rather than generalized central nervous system depression.

Among the receptor interactions of lurasidone, antagonism of the 5-HT7 receptor has generated particular interest because of its established role in circadian rhythm regulation and REM sleep physiology. The 5-HT7 receptor is highly expressed within the suprachiasmatic nucleus, the principal circadian pacemaker, while serotonergic projections from the midbrain raphe contribute to nonphotic phase resetting and synchronization of the sleep-wake cycle [11,12]. Experimental and genetic studies have further implicated the 5-HT7 receptor in REM sleep regulation and maintenance of normal sleep-wake cycling [11]. Consequently, antagonism of the 5-HT7 receptor represents one biologically plausible mechanism through which lurasidone may influence sleep beyond simple histamine-mediated sedation. However, lurasidone also exhibits pharmacologic activity at D2, 5-HT2A, and 5-HT1A receptors, and the available studies do not isolate 5-HT7 receptor antagonism as the specific mediator of its observed sleep effects. Accordingly, the contribution of 5-HT7 receptor antagonism remains a receptor-based hypothesis rather than an experimentally established mechanism. Direct evidence demonstrating clinically meaningful circadian modulation in humans also remains limited.

Preclinical evidence provides additional mechanistic support for this hypothesis. In a rodent model, Murai and colleagues evaluated seven adult male rats administered lurasidone (1, 3, or 10 mg/kg) at the start of the lights-on period and recorded sleep electroencephalography for six hours after dosing. Lurasidone shortened REM sleep duration, prolonged the mean duration of wakefulness and NREM sleep bouts, and increased slow-wave activity while reducing faster EEG frequencies during NREM sleep, findings consistent with more consolidated NREM sleep [14]. In contrast, the subsequent placebo-controlled human phase-advance study demonstrated improvements in sleep maintenance without significant changes in REM sleep [15]. These differing findings may reflect species-specific differences in sleep physiology, variations in experimental paradigms, dosing regimens, and outcome measures rather than contradictory pharmacologic effects. Although extrapolation from animal models to human sleep physiology should be made cautiously, these preclinical observations provide biologic plausibility for further clinical investigation.

Objective evidence evaluating the effects of lurasidone on sleep remains limited but encouraging. In a randomized, placebo-controlled crossover polysomnographic study, Krystal and Zammit evaluated 54 healthy volunteers who completed two treatment periods in randomized order, each consisting of two laboratory nights. On the second night of each period, bedtime was experimentally advanced by four hours, and participants received a single dose of lurasidone 40 mg or placebo. Compared with placebo, lurasidone significantly increased total sleep time by an average of 28.4 minutes (p<0.05), decreased wake after sleep onset and wake time after the final awakening, and increased sleep efficiency. No significant effects were observed on sleep onset, REM sleep, or slow-wave sleep [15]. Because the phase-advance paradigm incorporated experimentally advanced sleep scheduling, the findings suggest that lurasidone may improve sleep continuity under conditions of acute sleep schedule advancement. However, the study did not assess endogenous circadian phase, dim-light melatonin onset, core body temperature, phase-shifting responses, or other circadian biomarkers. Therefore, these findings should not be interpreted as direct evidence that lurasidone alters circadian physiology.

Clinical evidence in psychiatric populations remains comparatively sparse. In a post hoc analysis of a six-week randomized controlled trial involving children and adolescents with bipolar depression, Singh and colleagues evaluated 343 participants randomized to flexibly dosed lurasidone (20-80 mg/day) or placebo [16]. Compared with placebo, lurasidone was associated with significant improvements in sleep disturbance that accompanied overall clinical improvement. However, sleep outcomes were evaluated using symptom-based clinical measures rather than polysomnography, actigraphy, or circadian biomarkers. Accordingly, it remains uncertain whether these improvements reflected direct modulation of sleep continuity or sleep architecture or secondary benefits associated with successful treatment of the underlying mood disorder. Nevertheless, preliminary human evidence suggests that lurasidone may improve sleep continuity without pronounced antihistaminergic sedation, whereas preclinical evidence suggests possible effects on REM and NREM sleep [7,14-16]. Its activity at 5-HT7 and 5-HT2A receptors provides biologic plausibility for these observations, although the available studies do not establish either receptor as the specific mediator of the reported sleep effects.

Taken together, the available evidence suggests that lurasidone may improve sleep continuity with relatively little intrinsic sedation. Preclinical findings suggest possible effects on REM and NREM sleep, whereas preliminary clinical evidence suggests improvements in sleep continuity without consistent changes in REM sleep or direct evidence of circadian modulation [7,14-16]. These conclusions should be interpreted cautiously given the small number of dedicated sleep studies and the heterogeneity of the available evidence.

Comparison with other second-generation antipsychotics

The effects of second-generation antipsychotics on sleep vary considerably according to receptor-binding profile, patient population, and the specific sleep outcome being examined. Some agents improve sleep initiation or continuity primarily through sedation, whereas others may alter slow-wave sleep, REM sleep, or circadian regulation through serotonergic, dopaminergic, and adrenergic mechanisms. Moreover, an increase in total sleep time does not necessarily indicate normalization of sleep architecture or improved next-day functioning. Comparisons among agents must therefore distinguish subjective somnolence from objective changes in sleep continuity and sleep-stage distribution [1,7,8,10].

Clozapine and olanzapine are among the more sedating second-generation antipsychotics because of their substantial affinity for histamine H1, muscarinic M1, and α1-adrenergic receptors. Clozapine has generally been associated with shorter sleep latency and improvements in total sleep time and sleep efficiency in patients with schizophrenia, although its pronounced sedative effects may also contribute to excessive sleep duration and daytime somnolence [7,8,10]. Olanzapine has demonstrated comparatively consistent effects on objective sleep measures, including increased total sleep time, sleep efficiency, and slow-wave sleep [10,17]. However, olanzapine may not normalize all aspects of sleep physiology. Göder and colleagues found that although olanzapine increased slow-wave sleep, it decreased sleep-spindle density, illustrating that improvement in one component of sleep architecture may occur alongside disruption of another [18].

Quetiapine also has substantial H1 and α1-adrenergic receptor antagonism and is commonly associated with sedation. In healthy volunteers, low doses of quetiapine improved sleep initiation and continuity, increased total sleep time and sleep efficiency, and increased stage N2 sleep under both normal and experimentally disturbed sleep conditions [19]. However, findings in clinical populations have been less consistent. Studies involving patients with schizophrenia or mood disorders have not uniformly demonstrated sustained improvements in sleep efficiency or architecture, and some have reported increased sleep latency, wakefulness after sleep onset, and REM sleep latency with reductions in slow-wave and REM sleep [10,20]. These findings suggest that the sleep-promoting effects observed in healthy volunteers may not translate directly to patients with psychiatric illness. Furthermore, the sedative effects of quetiapine may produce residual daytime sleepiness or impaired vigilance, reinforcing the distinction between facilitating sleep and improving restorative sleep without next-day impairment [7,8].

Risperidone appears to have less consistent effects on sleep. Its lower affinity for histamine H1 and muscarinic receptors generally produces less sedation than clozapine, olanzapine, or quetiapine, but polysomnographic studies have not identified a uniform pattern of improvement in sleep continuity or sleep architecture [1,10]. Paliperidone, the principal active metabolite of risperidone, has shown somewhat more favorable effects in selected studies, including reductions in sleep latency and increases in total sleep time, sleep efficiency, slow-wave sleep, and REM sleep. Nevertheless, the limited number and heterogeneous design of these investigations prevent firm conclusions regarding its relative sleep-promoting properties [10].

Aripiprazole differs from the more sedating agents because of its partial agonism at dopamine D2 and 5-HT1A receptors and its relatively low affinity for histamine H1 and muscarinic M1 receptors. This receptor profile generally confers a lower propensity for somnolence but may produce activation, insomnia, or akathisia in susceptible patients [1,8]. Consequently, a low sedative burden should not automatically be interpreted as a beneficial effect on sleep. For some patients, reduced daytime somnolence may be advantageous, whereas for others, medication-related activation or restlessness may impair sleep initiation and continuity. Dedicated polysomnographic evidence examining the effects of aripiprazole on sleep remains limited compared with the evidence available for clozapine, olanzapine, and quetiapine [10].

In comparison, lurasidone has minimal affinity for histamine H1 and muscarinic M1 receptors and therefore lacks the pronounced antihistaminergic and anticholinergic sedation associated with clozapine, olanzapine, and quetiapine [1,13]. Nevertheless, preliminary human evidence suggests that lurasidone may improve sleep continuity without pronounced antihistaminergic sedation, whereas preclinical evidence suggests possible effects on REM and NREM sleep [14,15]. Its activity at 5-HT7 and 5-HT2A receptors provides biologic plausibility for these observations, although the available studies do not establish either receptor as the specific mediator of the reported sleep effects.

The available evidence therefore suggests that lurasidone may occupy an intermediate position between strongly sedating agents and more activating antipsychotics. Clozapine, olanzapine, and quetiapine may be advantageous when substantial sedation or improvement in sleep initiation is clinically desirable, but their benefits must be weighed against residual somnolence, metabolic adverse effects, and possible disruption of selected components of sleep architecture. Aripiprazole generally produces less sedation but may worsen insomnia or restlessness in some patients. Lurasidone may offer a comparatively lower sedative burden while retaining the potential to improve sleep continuity [7,8,10,13-15].

However, direct comparative conclusions should remain cautious. The available studies differ substantially in psychiatric diagnosis, baseline sleep disturbance, medication dose, treatment duration, and the inclusion of healthy volunteers versus clinical populations. Sleep may also improve indirectly through treatment of psychosis, depression, anxiety, or behavioral activation rather than through a direct effect on sleep-regulatory systems. Adequately powered head-to-head studies incorporating polysomnography, actigraphy, circadian biomarkers, subjective sleep quality, and next-day functional outcomes are needed to determine whether lurasidone provides a meaningful sleep-related advantage over other second-generation antipsychotics [7,8].

The receptor-binding profiles, relative sedative burden, and reported effects on sleep of selected second-generation antipsychotics are summarized in Table 2.

**Table 2:** Comparison of receptor-binding profiles, relative sedative burden, and reported effects on sleep among selected second-generation antipsychotics Receptor-binding profiles, relative sedative burden, and reported effects on sleep are summarized qualitatively from the cited literature and are intended to provide a comparative overview rather than quantitative receptor affinity data. Reported sleep effects may vary according to psychiatric diagnosis, medication dosage, treatment duration, concomitant therapies, and study methodology. Relative sedative burden reflects the propensity for clinically observed sedation and should not be interpreted as an indicator of improved physiologic sleep or normalization of sleep architecture. 5-HT, 5-hydroxytryptamine; D2, dopamine D2 receptor; H1, histamine H1 receptor; NREM, non-rapid eye movement; REM, rapid eye movement.

Antipsychotic	Key receptor-binding profile	Relative sedative burden	Reported effects on sleep
Clozapine	Strong H1, 5-HT2A, muscarinic M1, and α1-adrenergic receptor antagonism	Very high	Generally reduces sleep latency and improves total sleep time and sleep efficiency; excessive sedation and daytime somnolence may limit clinical utility [7,8,10].
Olanzapine	Strong H1 and 5-HT2A receptor antagonism with muscarinic activity	High	Consistently increases total sleep time, sleep efficiency, and slow-wave sleep; may reduce sleep-spindle density despite improvements in selected sleep parameters [10,17,18]
Quetiapine	Strong H1 and 5-HT2A receptor antagonism with α1-adrenergic receptor blockade	High	Improves sleep initiation and continuity; objective effects on sleep architecture remain variable across healthy volunteers and psychiatric populations [10,19,20].
Risperidone	Strong D2 and 5-HT2A receptor antagonism with relatively weak H1 receptor activity	Low to moderate	May improve sleep continuity, although objective polysomnographic findings remain inconsistent and no uniform pattern of sleep architecture improvement has been demonstrated [10]
Aripiprazole	Partial agonism at D2 and 5-HT1A receptors with 5-HT2A receptor antagonism and minimal H1 receptor affinity	Low	Lower propensity for sedation but may be associated with insomnia, activation, or akathisia in susceptible patients [8,10]
Lurasidone	D2 and 5-HT2A receptor antagonism; high affinity for 5-HT7 receptors; 5-HT1A partial agonism; minimal H1 and muscarinic M1 receptor affinity	Low	Preliminary evidence suggests improved sleep continuity with relatively little intrinsic sedation; possible effects on REM and NREM sleep require confirmation in larger clinical studies [7,13-16].

Clinical implications and future directions

Sleep disturbance is highly prevalent in schizophrenia, bipolar disorder, and other psychiatric illnesses, making sleep an important consideration when selecting an antipsychotic [2,5]. However, improving sleep should not be equated with simply producing sedation. The ideal agent would improve sleep continuity while minimizing residual daytime somnolence and preserving cognitive and functional performance.

Lurasidone may represent such an alternative because of its minimal affinity for histamine H1 and muscarinic M1 receptors, resulting in a relatively low intrinsic sedative burden [1,8]. Preliminary evidence suggests that lurasidone may improve sleep continuity through mechanisms involving 5-HT7 and 5-HT2A receptor antagonism rather than antihistaminergic sedation, although this hypothesis remains incompletely established [10,11,13]. However, low H1 receptor affinity does not preclude clinically relevant sleep-related adverse effects. Somnolence, insomnia, akathisia, and extrapyramidal symptoms have all been reported during lurasidone treatment and may influence both sleep quality and daytime functioning [1]. The occurrence and severity of these adverse effects may vary according to dose, timing of administration, and individual patient susceptibility. Although lurasidone's pharmacologic profile may be advantageous for patients who require preservation of daytime alertness, current evidence is insufficient to support its use as a primary treatment for insomnia or circadian rhythm sleep-wake disorders.

The available literature remains limited by small sample sizes, heterogeneous study populations, and the lack of long-term polysomnographic and circadian outcome measures. Future research should include larger prospective comparative studies and randomized head-to-head trials with other second-generation antipsychotics, incorporating standardized polysomnographic outcome measures, actigraphy, circadian biomarkers, and validated assessments of daytime functioning. Such studies will be necessary to better define the long-term effects of lurasidone on sleep architecture and determine whether it offers clinically meaningful advantages in sleep quality, circadian regulation, and daytime functioning beyond improvements attributable to treatment of the underlying psychiatric illness.

## Conclusions

Sleep disturbances are common across psychiatric disorders and contribute substantially to symptom burden, functional impairment, and reduced quality of life. Although many second-generation antipsychotics influence sleep, their effects differ considerably according to receptor-binding profile and should not be viewed solely through the lens of sedation. Improving sleep continuity and preserving next-day functioning are important therapeutic considerations that should be distinguished from direct modification of sleep architecture.

Lurasidone possesses a distinctive pharmacologic profile characterized by antagonism at the D2, 5-HT2A, and 5-HT7 receptors, with minimal histamine H1 and muscarinic M1 receptor affinity. Available human evidence suggests that lurasidone may improve sleep continuity while maintaining a relatively low intrinsic sedative burden, whereas preclinical studies suggest possible effects on REM sleep. However, the current evidence remains limited, and these findings have not been consistently demonstrated across species or experimental models. Overall, lurasidone represents a promising second-generation antipsychotic for patients in whom preservation of daytime alertness is an important clinical consideration. Nevertheless, the available evidence is based on a limited number of dedicated sleep studies and should be interpreted cautiously until confirmed in larger, well-designed comparative trials. Future investigations incorporating standardized polysomnography, actigraphy, circadian biomarkers, patient-reported sleep outcomes, and validated assessments of daytime functioning will be essential to determine whether lurasidone provides clinically meaningful advantages in sleep continuity, sleep architecture, and overall functional outcomes beyond improvements attributable to treatment of the underlying psychiatric illness.
